# Academic stress in adolescents: findings from a school-based study in Belagavi district

**DOI:** 10.3389/fpubh.2025.1631136

**Published:** 2025-11-03

**Authors:** Shivani Haritay, Mubashir Angolkar, Vinayak Koparde, Deshna Oswal, Alex Carvalho

**Affiliations:** ^1^Department of Public Health, KLE Jawaharlal Nehru Medical College, Belagavi, Karnataka, India; ^2^Department of Psychiatry, KLE Jawaharlal Nehru Medical College, Belagavi, Karnataka, India; ^3^Department of Epidemiology and Biostatistics, KLE University, Belagavi, Karnataka, India

**Keywords:** academic stress, adolescents, school, students, India, educational stress, ESSA

## Abstract

**Background:**

Academic stress has emerged as a significant risk factor for mental health development during adolescence. Schools have a considerable influence on adolescents’ development, but increasing academic pressures and social expectations have caused students to experience higher levels of stress, impacting their mental health and overall development.

**Objective:**

To assess academic stress levels and the factors associated with it among students aged 13–15 years in Belagavi district, Karnataka.

**Methods:**

A school-based survey was conducted among 1,426 students from four CBSE schools using universal sampling. Data were collected from July to September 2023. The Educational Stress Scale for Adolescents (ESSA) was used to measure academic stress. Statistical analysis involved Chi-square tests, Fisher’s exact test, and independent *t*-tests.

**Results:**

Among the 1,426 students, 74% reported high levels of academic stress, with 17% reporting medium levels. Academic stress levels were significantly associated with age (*p* = 0.027), area (*p* = 0.000), father’s education (*p* = 0.023), and gender (*p* = 0.001). Male students experienced significantly higher stress levels in study pressure, grade-related anxiety, self-expectation, and self-despondency (*p* < 0.05). Female students experienced slightly higher stress related to workload, though this was not statistically significant.

**Conclusion:**

Academic stress is prevalent among students and is significantly influenced by various sociodemographic variables.

## Introduction

Childhood and adolescence are crucial phases for mental health development, with the surrounding environment playing a vital role in shaping overall wellbeing and growth (1). Mental health issues among children and adolescents are a global concern, ranking among the primary causes of death, illness, and disability, particularly in older adolescents. Approximately 50% of mental health disorders begin by age 14, yet the majority remain undiagnosed and untreated (2).

Globally, systematic reviews have highlighted the adverse impact of academic stress on adolescent mental health. A recent review of 52 studies conducted across diverse countries reported that the majority demonstrated a positive association between academic pressure and problems such as anxiety, depression, and even suicidal ideation. Evidence further indicates that stress intensifies in the final years of schooling, with around one in six students experiencing excessive distress (3, 4).

India is home to one-fifth of the global adolescent and youth population, comprising approximately 30% of the country’s total population. According to the National Mental Health Survey 2016, 7.3% of teenagers experienced mental health conditions, with an equal distribution among boys and girls. A study reported that 23.33% of children and teenagers in Indian schools and 6.46% in communities experienced mental health issues (5–8).

Schools, where children spend a substantial portion of their time, have a significant influence on multiple aspects of development. These institutions contribute to shaping peer relationships, social interactions, academic achievement, emotional regulation, and behavioral and moral growth, all of which are closely linked to mental health (9). Consequently, academic stress has emerged as a prominent risk factor for adolescent mental health, with schools being a focal point for both the pressures and opportunities shaping their psychological wellbeing (10).

The growing concern of academic stress, driven by academic demands, personal factors, and social influences, disrupts students’ lives in numerous ways. It impacts their self-esteem, motivation, and behavior, often resulting in an inability to cope with academic challenges and withdrawal from other responsibilities (11). Students face constant pressure to achieve academic success, meet societal expectations, and attain personal goals, some of which may be overly ambitious or unattainable. This intensifies their stress levels, further negatively affecting their mental health (12).

This study is grounded in the Transactional Model of Stress and Coping [TMSC] in which primary and secondary appraisals shape stress response. In the school context, academic stress emerges from a continuous transaction between students and their environment (13). Transactional Model explains how students appraise these conditions—first whether the conditions are challenging [primary appraisal] and second whether they possess sufficient coping resources [secondary appraisal]. Academic Stress increases when perceived school demands outweigh available resources and coping [problem-focused or emotion -focused] is insufficient. Thus, the mechanism is—School environment—[demands, resources]—Appraisal [threat/resources]—coping—academic stress. This study sought to address two key questions—1. What are the levels of academic stress as measured by the Educational Stress Scale for Adolescents [ESSA] and 2. Which sociodemographic characteristics are associated with academic stress levels.

## Methods

### Study design, area, and period

Eligibility for the school survey analysis was based on a larger randomized controlled mental health intervention trial (RCT) (Clinical Trials Registry of India, CTRI Reg.no CTRI/2023/12/060663). The parent RCT targeted adolescents aged 13–15 years old adolescents, so students in classes 7th–9th from selected Central Board of Secondary Education (CBSE) schools were screened. The study protocol was approved by the KAHER Ethics Committee on Human Subjects for the Ph.D. Research Project (Ref. No. KAHER/EC/22-23/125-2). Data for the current analysis were collected between July and September 2023 in Belagavi district, Karnataka, India.

### Population inclusion and exclusion criteria

Students of class seven to ninth who were present on the day of data collection. Those who provided assent, in addition to parental consent and were willing to participate in the study period were included in the study. Students with severe hearing, visual, or communication impairments were excluded.

### Sample size determination and sample procedure

Four CBSE schools were selected using purposive sampling, and out of 1,620 students a total of 1,426 students were eligible and were included in the study:

**Table:** 

School	Students included		Total Strength
1	413	7th, 8th, 9th, each class had three divisions with 45 students in a division	405
2	612	7th, 8th, 9th, each class had five divisions each with 45 students in a division	675
3	233	7th, 8th, 9th, each class had two divisions each with 45 students in a division	270
4	168	7th, 8th, 9th, each class had two divisions each with 45 students in a division	270
	1,426		1,620

In school one, in class 9, eight students were repeaters.

### Data collection instrument and procedure

The first section of the questionnaire included sociodemographic characteristics that provided background information on students and their parents (Table 1). Second section of the questionnaire included the ESSA instrument used to assess academic stress among the students.

**Table 1:** Description of independent variables for study analysis.

Variable description		
Level	Description	Categorization
School level	Central Board of Secondary Education [CBSE]	CBSE school 1, 2, 3, 4
	Age In years	13, 14, 15
	Gender	Female or male
	Class	7th, 8th, 9th
Household level	Area [where the student resides]	Urban, Rural
	Family type	Nuclear [where only parents with their unmarried children stay together], Joint [a household where the nuclear family co-resides with additional adult relatives (e.g., paternal grandparents and/or father’s brother (paternal uncle) and his family) sharing the same household and kitchen.]
	Caste	General, OBC [OBC–Other Backward Classes], SC/ST [Scheduled caste/Scheduled tribe]
	Religion	Hindus, non-Hindus [includes Muslim, Christian, Jain, and Buddhist]
Education level	Parents [Mother and Father]	Secondary School Leaving Certificate [SSLC]/10th standard, Pre-University Course [PUC], Graduation, Post-graduation
Occupation level	Parents [Mother and Father]	Defense, Homemaker, Government Job, Private job

Formal permission to use The Educational Stress Scale for Adolescents (ESSA) was obtained from the author. Developed by Sun et al. (28), the ESSA is standardized and validated tool, including validation in studies conducted in India. In the present study, the scale was administered in its original language (English), so no local translation was required.

The ESSA consists of 16 items rated on five-point Likert scale, (16–80) with higher scores indicating greater academic stress. It assesses five factors: Pressure from Study (four items on learning pressure, peer competition, and concerns about the future), Workload (three items on homework and exams), Worry about Grades (three items on grade-related stress), Self-Expectation Stress (three items on unmet expectations), Despondency (three items on academic dissatisfaction) The scale has good internal consistency, with Cronbach’s alpha for the total scale at 0.82 and factor reliabilities ranging from 0.64 to 0.79.

### Data entry and statistical analysis

Data were cleaned, coded and entered in MS-Excel and exported to IBM Statistical Package for the Social Sciences (SPSS) version 20.0 for analysis. Sociodemographic data and academic stress levels were summarized by computing frequency and percentages. Academic stress categories were defined based on cut-off score validated in an Indian sample (14).

- Low: Scores −16 to 21
- Medium: Scores 22–42
- High: Scores 42 and above

Associations between academic stress levels and sociodemographic characteristics were assessed using Chi-square tests, with Fisher’s exact test applied when more than 20% of the expected cell count were less than five. Statistical significance was set at a *p*-value of < 0.05. Differences in mean scores of academic stress-related factors by gender were assessed using independent *t*-tests, and the assumptions of normality were verified; results were confirmed with Mann–Whitney U tests where appropriate. Multinomial logistic regression was used to identify sociodemographic predictors of academic stress.

### Ethical consideration

Before data collection the purpose of the study was clarified to the principal and students of each school. Permission was obtained from the heads of all four schools. Assent and consent were obtained from the students and their parents.

## Results

**Figure 1:**
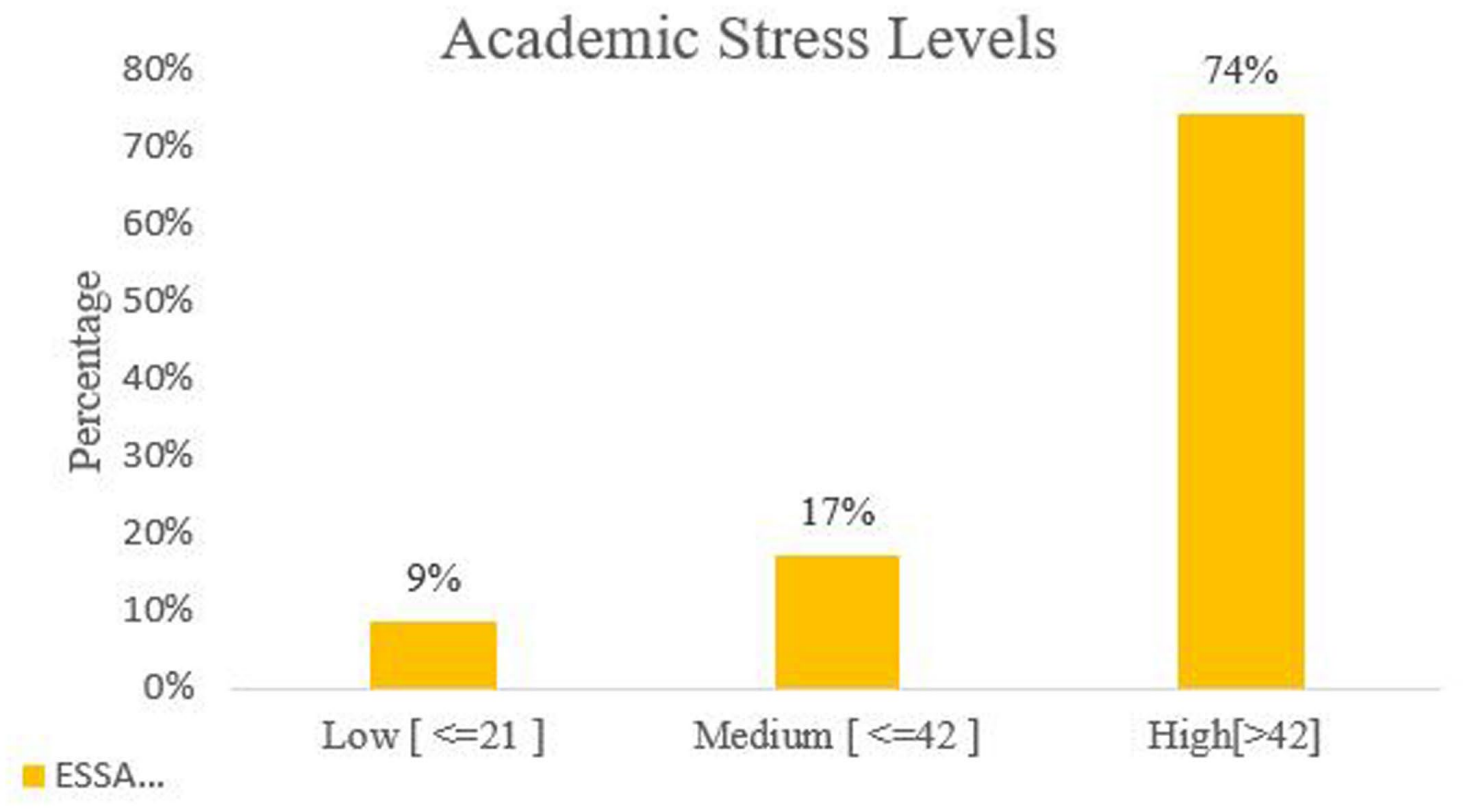
Academic stress levels among students.

Figure 1 shows the overall academic stress levels among students. It was observed that 74% of students had scored above 42, indicating high levels of academic stress, followed by 17% of students who scored ≤42, indicating medium levels of academic stress.

Table 2 provides the basic characteristics of the school students. Among the 1,426 students, the largest number (69.1%) were 13 years old, with 57.2% coming from urban areas and 82.5% from nuclear families. Most participants’ parents, both fathers (72.2%) and mothers (79.2%), had completed schooling up to the 10th grade. Among the students, 46.1% were female and 53.9% were male. In terms of religion, 88.1% were Hindu. Regarding parents’ occupations, 54.4% of fathers were employed in Defense, and 90.6% of mothers were homemakers.

**Table 2:** Socio-demographic characteristics of the students (*N* = 1,426).

Variable	Category	% (*n*)
School	1	29.0% (413)
	2	42.9% (612)
	3	16.3% (233)
	4	11.8% (168)
Class	7th	34.9% (497)
	8th	30.1% (429)
	9th	35.1% (500)
Age (years)	13	69.1% (985)
	14	26.1% (372)
	15	4.8% (69)
Gender	Female	46.1% (658)
	Male	53.9% (768)
Caste	General	77.8% (1,110)
	OBC	10.0% (143)
	SC/ST	12.1% (173)
Religion	Hindu	88.1% (1,257)
	Non-Hindu	11.8% (169)
	Muslim	7.0% (100)
	Christian	0.9% (13)
	Jain & Buddhist	3.9% (56)
Mother’s occupation	Homemaker	90.6% (1,292)
	Government job	8.7% (124)
	Private job	0.7% (10)
Father’s occupation	Defense	54.4% (776)
	Government job	12.7% (181)
	Private job	32.9% (469)
Area of residence	Rural	42.8% (611)
	Urban	57.2% (815)
Family type	Nuclear	82.5% (1,177)
	Joint	17.5% (249)
Mother’s education	School	79.2% (1,129)
	PUC	12.1% (173)
	Graduation	6.9% (99)
	Post-graduation	1.8% (25)
Father’s education	School	72.2% (1,029)
	PUC	15.6% (223)
	Graduation	9.3% (132)
	Post-graduation	2.9% (42)

OBC, Other Backward Classes; SC/ST, Scheduled caste/Scheduled tribe; PUC, Pre-University College.

Table 3 presents the distribution of academic stress levels among students across various sociodemographic characteristics using Chi-square and Fisher’s exact test. Among the various sociodemographic variables assessed, age (*p* = 0.027), area (*p* = 0.000), father’s education (*p* = 0.023), and gender (*p* = 0.001) were significantly associated with academic stress levels.

**Table 3:** Association of academic stress levels with sociodemographic characteristics.

Sociodemographic characteristics		Academic stress levels			Chi-Sq.	Significance
		Low (%)	Medium (%)	High (%)		
Age	13	82 (8.3)	180 (18.3)	723 (73.4)	10.996	0.027*
	14	29 (7.8)	53 (14.2)	290 (78.0)		
	15	12 (17.4)	13 (18.8)	44 (63.8)		
Area	Rural	99 (16.2)	111 (18.2)	401 (65.6)	82.088	0.000*
	Urban	22 (2.9)	135 (16.6)	656 (80.5)		
Family type	Nuclear	104 (8.8)	207 (17.6)	866 (73.6)	1.059	0.589
	Joint	19 (7.6)	39 (15.7)	191 (76.7)		
Father education	School	92 (8.9)	172 (16.7)	765 (74.3)	14.678	0.023*
	PUC	21 (9.4)	33 (14.8)	169 (75.8)		
	Graduation	5 (3.8)	27 (20.5)	100 (75.8)		
	Post-graduation	5 (11.9)	14 (33.3)	23 (54.8)		
Father occupation	Defense	73 (9.4)	129 (16.6)	574 (74.0)	3.888	0.421
	Government job	17 (9.4)	37 (20.4)	127 (70.2)		
	Private job	33 (7.0)	80 (17.1)	356 (75.9)		
Gender	Female	40 (6.1)	102 (15.5)	516 (78.4)	14.395	0.001*
	Male	83 (10.8)	144 (18.8)	541 (70.4)		
Mother education	School	102 (9.0)	188 (16.7)	839 (74.3)	9.859 ^a^	0.118
	PUC	12 (6.9)	29 (16.8)	132 (76.3)		
	Graduation	5 (5.1)	21 (21.2)	73 (73.7)		
	Post-graduation	4 (16.0)	8 (32.0)	13 (52.0)		
Mother occupation	Homemaker	116 (9.0)	215 (16.6)	961 (74.4)	5.770 ^a^	0.180
	Government job	6 (4.8)	29 (23.4)	89 (71.8)		
	Private job	1 (10.0)	2 (20.0)	7 (70.0)		
Religion	Hindu	112 (8.9)	212 (16.9)	933 (74.2)	3.810 ^a^	0.683
	Non-Hindu	11 (6.5)	34 (20.1)	124 (73.4)		

*Significant at <5% level, Fisher’s Exact^a^.

OBC, Other Backward Classes; SC/ST, Scheduled caste/Scheduled tribe; PUC, Pre-University College.

Table 4 presents the results of the independent *t*-test for academic stress factors by gender. Independent *t*-tests were applied to compare gender differences in academic stress across five categories. Male students exhibited significantly higher stress levels compared to female students in study pressure (*M* = 13.45, *t* = 2.649, *p* < 0.05), grade-related anxiety (*M* = 11.25, *t* = 4.386, *p* < 0.05), self-expectation (*M* = 10.03, *t* = 4.218, *p* < 0.05), and self-despondency (*M* = 9.27, *t* = 2.906, *p* < 0.05). On the other hand, female students reported slightly higher stress related to workload (*M* = 9.33) compared to male students (*M* = 9.20), though this difference was not statistically significant (*t* = −0.848). These results suggest that while academic stress affected both genders, male students reported significantly greater mean scores in academic stress categories.

**Table 4:** Results of independent *t*-test comparing academic stress categories between male and female students.

Academic stress categories	Female [*N* = 655]	Male [*N* = 763]	*t*	*p*-value
	Mean ± SD	Mean ± SD		
Pressure from study	13.45 ± 3.51	12.95 ± 3.56	2.649	0.008*
Workload	9.2 ± 2.87	9.33 ± 3.14	−0.848	0.397
Worry about grades	11.25 ± 2.63	10.61 ± 2.77	4.386	0.001*
Self-despondency	9.27 ± 2.96	8.82 ± 2.88	2.906	0.004*
Self-expectation	10.03 ± 2.87	9.38 ± 2.93	4.218	0.001*

*Significant at <5% level, *N* = total number of students, M = Mean, SD = Std. Deviation.

Table 5 shows that the regression model was statistically significant (LR *χ*^2^ (40) = 247.30, *p* < 0.001) with Pseudo-*R*^2^ of 0.118, indicating that the sociodemographic characteristics accounted for 12% of the variation in academic stress. Academic stress levels increased sharply with grade progression. Compared with 7th graders, students in 8th grade were over 19 times more likely to report medium academic stress level (RRR = 19.26, 95% CI: 7.53–49.26, *p* < 0.001) and almost 23 times more likely to report high academic stress level (RRR = 22.97, 95% CI: 9.29–56.82, *p* < 0.001). Those in 9th grade also showed elevated risks, being three times more likely to experience medium stress (Medium: RRR = 3.00, 95% CI: 1.39–6.45; High: RRR = 4.85, 95% CI: 2.42–9.73). Place of residence was another strong predictor. Urban students were six times more likely to report medium stress and nearly 10 times more likely to report high stress compared with rural students (Medium: RRR = 6.30, 95% CI: 3.64–10.91; High: RRR = 9.74, 95% CI: 5.88–16.16; both *p* < 0.001). Gender differences were also evident, male students had significantly lower odds of reporting both medium and high stress compared with females (RRR = 0.59, 95% CI: 0.36–0.97, *p* = 0.037) or High stress categories (RRR = 0.42, 95% CI: 0.27–0.65, *p* < 0.001). Caste and family structure showed more specific associations. Students from OBC backgrounds and those from joint families were significantly more likely to experience high stress compared with their peers. High stress, OBC caste (RRR = 2.43, 95% CI: 1.10–5.38, *p* = 0.028) and joint family type (RRR = 2.07, 95% CI: 1.13–3.76, *p* = 0.018). Other variables including religion, parental education, parental occupation was not independently associated with academic stress levels after adjustment.

**Table 5:** Multinomial logistic regression of sociodemographic predictors of academic stress levels among adolescents (*N* = 1,426).

Predictor (reference group)	Category	Medium vs. Low RRR (95% CI)	High vs. Low RRR (95% CI)
Class (7th)	8th	19.26 (7.53–49.26)***	22.97 (9.29–56.82)***
	9th	3.00 (1.39–6.45)**	4.85 (2.42–9.73)***
Age (13 years)	14 years	0.54 (0.26–1.15)	0.62 (0.31–1.22)
	15 years	0.39 (0.13–1.18)^†^	0.23 (0.09–0.60)**
Gender (Female)	Male	0.59 (0.36–0.97)*	0.42 (0.27–0.65)***
Caste (General)	OBC	1.40 (0.56–3.46)	2.43 (1.10–5.38)*
	SC/ST	1.40 (0.61–3.20)	1.64 (0.77–3.49)
Religion (Hindu)	Non-Hindu	1.40 (0.91–2.16)	1.27 (0.84–1.91)
Area (Rural)	Urban	6.30 (3.64–10.91)***	9.74 (5.88–16.16)***
Family type (Nuclear)	Joint	1.48 (0.76–2.90)	2.07 (1.13–3.76)*
Father’s education (SSLC)	PUC	0.77 (0.38–1.55)	0.86 (0.47–1.58)
	Graduation	2.70 (0.80–9.13)	2.33 (0.74–7.37)
	Post-graduation	1.29 (0.22–7.66)	0.49 (0.09–2.72)
Mother’s education (SSLC)	PUC	1.26 (0.55–2.88)	1.27 (0.61–2.66)
	Graduation	1.01 (0.27–3.80)	1.00 (0.29–3.45)
	Post-graduation	0.44 (0.06–3.32)	0.28 (0.04–1.92)
Father’s occupation (Defense)	Government job	0.79 (0.38–1.62)	0.62 (0.32–1.19)
	Private job	1.04 (0.59–1.82)	1.09 (0.66–1.81)
Mother’s occupation (Homemaker)	Government job	1.98 (0.73–5.36)	1.48 (0.57–3.83)
	Private job	2.23 (0.17–29.54)	2.89 (0.28–30.07)

Base outcome = Low stress, RRR, Relative Risk Ratio; CI, Confidence Interval, ^†^*p* < 0.10; *p < 0.05; **p < 0.01; ***p < 0.001.

## Discussion

This study assessed academic stress levels and associated factors among 1,426 (13–15 years) using the Educational Stress Scale for Adolescents (ESSA). We observed that 74% scored above 42 (high stress) and 17% fell in the medium range (Figure 1). In adjusted multinomial models (Table 5) grade progression (8th/9th vs. 7th) and urban residence were the strongest predictors of elevated stress, while boys had lower adjusted odds of medium/high academic stress than girls despite scoring higher than girls on specific academic stress factors (study pressure, grade anxiety, self-expectation, despondency) in *t*-tests (Table 4). Additional associations were observed for caste (OBC) and joint family structure showed positive associations with high academic stress, whereas parental education and occupation were not independently related to academic stress levels after the adjustment.

### How our prevalence compares and why it is high

The prevalence documented here exceeds compared to several earlier studies. For example, a work using ESSA along with KADS found only 22% of students in the high stress category (15), case–control studies have often restricted stress *t* cases while controls reported mild stress (16). Studies relying on SAAS or PAS scales typically show 31–48% of students experiencing high or moderate stress, often with subscale gender differences in workload or time restraints, but weaker overall associations with performance (17). A pattern replicated in newer Indian cohorts where overall sex differences may be small but subscale contrasts persist (18). In India, academic achievement is deeply entwined with parental expectations and exam based progression. Urban students, are embedded in environments where private tutoring, high stakes assessments, and peer competition are common. Yet school mental health support remains limited or uneven (19). Studies of competitive exam contexts similarly document high academic stress, high perceived parental pressure, and low wellbeing, are widespread across streams and classes when systematic pressure dominates (20). Together these structural features offer a plausible explanation for the particularly high levels observed in our study, especially in urban and higher-grade students.

### Interpreting sociodemographic variables through the transactional model

The Transactional Model of stress and coping (13) helps in understanding the sociodemographic patterns observed. Academic stress arises when the students appraise school demands such as exams, assignments and other activities exceed their available resources like time, support and coping strategies. For many, especially in urban settings and higher grades, these demands are perceived as threats rather than challenges. Contemporary evidence in younger secondary grades show that academic stress directly increases cognitive, emotional, and behavioral components of burnout, with anxiety acting as a key mediator, while self-efficacy buffers against these effects (21). Response surface work further reveal that when both academic pressure and psychological imbalance are high, the risk of depressive symptoms is amplified, with effects largest in pivotal exam years (22). This aligns with our multinomial findings, where 8th and 9th graders have higher odds of academic stress compared with their 7th grade peers, suggesting an early escalation of threat appraisals in the school settings.

### Gender–reconciling *t*-test and regression

Our gender findings highlight the complexity of academic stress experiences. First, boys scored higher than girls on study pressure, grade anxiety, self-expectation, and despondency (Table 4), consistent with factor-level sex contrasts reported on PAS and SAAS subscales (17, 18). Second, in the adjusted multinomial models, boys had lower odds of medium/high overall academic stress than girls. This contradiction is expected when domain specific elevations among boys do not translate into higher overall academic stress classifications once grade, urban residence and family factors are considered. Moreover, academic stress may manifest in divergent behavioral outcomes. For instance, recent work shows that social involvement can rise as a coping channel when academic emotions are low (23). These observations suggest that interventions should be sensitive to factor specific vulnerabilities—addressing grade related anxiety and self-expectations in boys while recognizing that, overall girls remain at higher risk for medium to high academic stress.

### Family/school processes vs. distal socioeconomic characteristics

While preliminary analyses hinted at associations between parental education and academic stress (Table 3), but these links decreased after adjustments. This is consistent with the evidence that proximal process—the nature of parental involvement and the quality of school climate are more influential than the distal socioeconomic markers. Supportive, autonomy–enhancing parental involvement has been shown to reduce internalizing symptoms, whereas parental pressure and psychological control tend to heighten stress, with the latter often exerting a stronger negative effect than the protective influence of involvement (24). School level factors such as classroom management. Teacher support, and school connectedness similarly shape adolescents’ experiences of stress and wellbeing (25). These insights help why parental education and occupation variables can lose explanatory power once grade, urban context and family functioning styles are accounted for.

### Mechanisms linking academic stress to mental health risks

Longitudinal evidence underscores the pathways through which academic stress translates into mental health problems. Sleep disturbances and loneliness have been identified as major mediators linking stress to depression and anxiety, while physical activity contributes modestly and inconsistently across outcomes (26). Importantly, time spent studying does not directly predict emotional outcomes but rather exerts its influence through student’s perceptions of stress, reinforcing the central role of appraisal in the Transactional Model (13). In or urban, high grade subgroup, where demands and comparison are dens, these mediators likely intensify the risk. There is also evidence that enhancing self-efficacy can weaken the stress [the stress → anxiety → burnout] (27) highlighting a need for interventions for schools.

### Positioning our findings in the wider Indian context

Narrative syntheses of Indian school mental health emphasize cultural valuation of grades, stigma of failure and heavy reliance on private tutoring with reports of substantial exam anxiety in board years (19). Studies in competitive exam cohorts likewise document high stress and high parental pressure across streams, with degraded wellbeing (20). Our findings that urban students with those in higher grades bear the greatest burden are consistent with these realities. The additional associations with caste and family structure likely reflect underlying social roles and household expectations that shape adolescents appraisals and coping opportunities. Disparities in prevalence estimates across studies such as lower rates reported with SAAS or PAS (15, 17) are best understood as function of differences in scale content, cut off, sample characteristics and timing, particularly with post-COVID-19 cohorts (15–18).

## Limitations

Among the several limitations of this study are: As this was a cross-sectional study, no casual or temporal relationships can be inferred from the study findings. The type of school [CBSE] and the purposive sampling of school limit the generalizability of findings to other school boards or government schools. The study involved self-reported scale on academic stress, no other psychological and social stressors were studied, which could interact with academic stress.

## Conclusion

This study reveals a high prevalence of academic stress among adolescents of CBSE schools, Belagavi, Karnataka, with grade progression and urban residence, being the strongest determinants. Female sex, OBC caste and joint family structure were also the significant predictors, underscoring how rising academic demands and limited coping resources contribute to stress. These findings interpreted through Transactional Model of stress and coping, highlight the role of school demands and social context in shaping stress among adolescents. It is recommended that management and the teachers need to sensitize the students the importance of school counselor and how they can be benefited from the counseling. Mental health club for Students should be formed so that students can actively participate in fun learning activities based on mental health.

## Data Availability

The original contributions presented in the study are included in the article/supplementary material, further inquiries can be directed to the corresponding author.
